# TB infection prevention and control at public health facilities in //Karas region, Namibia

**DOI:** 10.1017/ash.2025.10248

**Published:** 2025-12-16

**Authors:** Nicolett Muzuki Nyambe, Carolie Cloete, Andrit Lourens

**Affiliations:** 1 Department of Clinical Health Sciences, School of Health Sciences, Faculty of Health, Natural Resources and Applied Sciences, Namibia University of Science and Technology (NUST), Windhoek, Namibia; 2 Department of Environmental and Public Health, Ministry of Health and Social Services, Keetmanshoop, Namibia; 3 Division of Emergency Medicine, Department of Family, Community and Emergency Medicine, University of Cape Town (UCT), Cape Town, South Africa

## Abstract

**Objectives:**

To evaluate healthcare workers’ (HCWs) providing tuberculosis (TB) services knowledge, attitudes, and practices (KAP) regarding infection prevention and control (IPC), assess barriers and facilitators to TB IPC implementation and adherence, and evaluate existing TB IPC policies at public health facilities in Namibia’s //Karas region.

**Design:**

Concurrent mixed-methods design.

**Setting:**

Public healthcare facilities in Namibia’s //Karas region.

**Methods:**

Quantitative data were collected using an online questionnaire distributed via email and social media platforms to HCWs providing TB services. Descriptive statistics were used to summarize respondent characteristics and KAP scores, followed by bivariate analysis using the Pearson χ^2^ test (*P* < .05) to assess associations between knowledge scores and respondent characteristics. Focus group discussions (FGDs) were conducted with TB focal persons from District Coordinating Committees, transcribed, and analyzed thematically using a 6-phased approach. Data collection spanned 8 months (April 17–November 10, 2023).

**Results:**

HCWs demonstrated good knowledge (91.2%) and positive attitudes (85.3%) toward TB IPC, with practice scores less optimal (61.8%). Knowledge was associated with district (*P* = .001), department (*P* = .036), and education level (*P* = .010). Staff shortages were the most cited barrier, and training emerged as a key facilitator. FGDs confirmed the implementation of TB IPC policies at institutional level and revealed barriers, including inadequate infrastructure, limited resources, stigmatization, and lack of managerial support. Facilitators included effective communication, health education, and personal protective equipment availability.

**Conclusion:**

Strengthening TB IPC requires investment in infrastructure, training, consistent monitoring, and policy enforcement. Targeted IPC interventions can address key barriers and improve adherence across public health facilities.

## Introduction

Tuberculosis (TB), though preventable and curable, remains a major global health threat, regaining its position as the foremost infectious killer in 2023.^1^ In that year, an estimated 10.8 million people developed TB worldwide, with 1.25 million deaths, including 161,000 people with human immunodeficiency virus (HIV).^1^ South-East Asia and Africa continue to carry the highest TB-burden, with Namibia among the top 30 high-burden countries globally.^1^

Across Africa, weak health systems, limited healthcare access, and high HIV prevalence undermine TB control efforts.^2^ Namibia faces similar challenges, where the HIV-TB co-epidemic disproportionately affects marginalized and poor communities.^3^ Nonetheless, progress has been made, with a reduced case notification rate of 468 per 100,000 people in 2023.^4^

To curb TB transmission in healthcare settings, the World Health Organization recommends TB infection prevention and control (IPC) measures, including administrative controls, environmental measures, and personal protective equipment (PPE).^5^ Although Namibia’s national IPC policies support these measures, inconsistent implementation and knowledge gaps among healthcare workers (HCWs) persist.^6,7^

Although early studies revealed inadequate knowledge about TB IPC among HCWs,^8,9^ more recent evidence suggests an improvement in knowledge, alongside gaps between knowledge and actual practice.^10^ Trained HCWs and those with higher qualifications or formal TB IPC education generally demonstrate greater knowledge and improved TB IPC practices.^11^

Barriers to effective TB IPC include fragmented HIV/TB services, weak policies, limited funding and infrastructure, weak leadership, inadequate knowledge and training, low PPE use, stigma, poor risk perception, unsupportive work culture, and competing workloads.^12,13^ Facilitators include targeted training, supportive leadership, good communication, sufficient resources, and a strong safety culture.^12,13^ Evidence also suggests poor TB IPC implementation in high-burden settings often stems from the absence of policies, poor policy enforcement, or poorly informed HCWs, increasing their exposure to TB.^14,15^

In the //Karas region, a high TB-burden area in Namibia, no comprehensive assessment of HCWs’ knowledge, attitudes, and practices (KAP), barriers, and facilitators of TB IPC implementation and adherence has been conducted. Additionally, the practical effectiveness of IPC policies and potential policy gaps have not been assessed.

This study aimed to evaluate the TB IPC challenges at public health facilities in the //Karas region, Namibia. The specific objectives were to (1) evaluate HCWs’ providing TB services KAP regarding TB IPC, (2) assess the barriers and facilitators to the implementation and adherence to TB IPC procedures, and (3) evaluate policies aimed at TB IPC at public health facilities in the //Karas region.

## Methods

### Study design

We conducted a concurrent mixed-methods study in the //Karas region, Namibia. The quantitative phase involved a cross-sectional survey of the TB IPC KAP of HCWs providing TB services at public health facilities, as well as barriers and facilitators to TB IPC adherence (phase 1), while the qualitative phase comprised focus group discussions (FGDs) with TB focal persons from District Coordinating Committees (DCCs) of each district within the region (phase 2).

### Study setting

//Karas is Namibia’s largest region in the South, covering 161,514 km^2^, with a population of 109,893, and 3 districts (Keetmanshoop, Lüderitz, and Karasburg).^16,17^ The health and social work industry employs around 672 people.^18^ The region’s public health system comprises district hospitals, clinics, and health centers that refer patients to intermediate and specialist hospitals in the capital, Windhoek. The region has 23 public health facilities under the Ministry of Health and Social Services (MoHSS), but only 15 provide TB services.^19^ Each district hospital’s DCC plays a key role in implementing the National TB and Leprosy Programme. The DCC also oversees health management, planning, and coordination within the district, ensuring the implementation of TB IPC measures in the public and private sectors.

### Study population and sampling

For the quantitative phase, eligible participants were HCWs (eg, doctors, nurses, environmental health personnel, and other HCWs) involved in TB services at public health facilities in the //Karas region employed for > 6 months. HCWs with < 6 months’ experience at their current facility, those on leave during data collection, and hospital staff involved in administration and management were excluded.

FGD participants were TB focal individuals from DCCs, including Senior Medical Officers, primary healthcare (PHC) supervisors, District TB and Leprosy Coordinators (DTLCs), and Heads of Departments (HoDs) of each district hospital in //Karas region. Private health supervisors, HoDs, and managers or executives of non-governmental organizations were excluded.

Non-probability sampling techniques were employed for both study phases, convenience sampling for the quantitative phase and purposive sampling for the qualitative phase. A sample size of 203 was calculated for the quantitative phase, targeting a 95% confidence level (CI) and a 3% margin of error, with an estimated population size of 250 HCWs. However, 102 responses were obtained, resulting in a 7% margin of error. For the qualitative phase, each FGD comprised 5–8 participants, conducted separately in the 3 districts.

### Data collection

An online KAP questionnaire was developed in English using Google Forms (Supplemental File 1). The questionnaire consisted mostly of closed-ended questions and was designed to assess TB IPC-related KAP among HCWs. The questions were informed by the MoHSS TB IPC Guideline 2021,^20^ National Guidelines for the Management of Tuberculosis 4^th^ Edition 2019,^21^ and some were adapted from a similar study.^22^

Knowledge was operationalized through 10 multiple-choice questions (true, do not know, false) assessing respondents’ understanding of TB transmission, symptoms, IPC measures, and relevant guidelines. Scores were calculated based on the number of correct responses, with higher scores indicating greater knowledge. Attitudes were measured using a 3-point Likert scale (agree, neutral, disagree) with 10 questions gauging respondents’ beliefs, perceptions, and willingness to implement TB IPC measures. Practices were measured using a 3-point Likert scale (always, sometimes, never) with 20 questions evaluating the implementation of IPC (administrative, environmental, and personal protective control) measures in practice. Barriers and facilitators were measured through open-ended questions exploring factors influencing adherence to TB IPC procedures.

The questionnaire was reviewed by 2 TB experts, the Karas Regional TB Leprosy Coordinator and a senior lecturer from the Namibia University of Science and Technology (NUST). The online questionnaire was pilot tested by 10 HCWs providing TB services within the region, who were excluded from the main study. Drawing on the expert input and the pilot test, the survey was revised and refined before data collection.

The questionnaire was emailed to HoDs at public health facilities for distribution through email or digital communication groups. Reminder emails were sent biweekly.

Three FGDs were conducted with DCC members from each district. An FGD guide (Supplemental File 2) was used to evaluate TB IPC policies and identify barriers and facilitators to their implementation. Invitation emails were sent, and written consent was obtained prior. FGDs were moderated by the researcher, audio-recorded, and notes taken by a research assistant. Data collection spanned 8 months (April 17–November 10, 2023).

To ensure credibility, the researcher employed discussion techniques during FGDs, rephrasing and clarifying questions to elicit clear and consistent responses, which increased participant engagement. Transferability was supported through a detailed description of the study context, population, FGD process, and findings, including participant quotations. Dependability was achieved by ensuring error-free transcription accurately reflecting participants’ responses. Confirmability was maintained through reflexivity, with the researcher taking reflexive notes during FGDs to minimize bias. To reduce respondent bias, discussion points were shared in advance, allowing participants to prepare thoughtfully.

### Data analysis

Questionnaire data were extracted and cleaned in Excel and imported into IBM SPSS for Windows Version 28^23^ for analysis with support from a statistician. Descriptive statistics were used to summarize KAP responses as well as report barriers and facilitators.

KAP responses were scored using the method described by Akande.^24^ Correct knowledge responses scored 1; incorrect or “don’t know” scored 0. Scores ≥ 60% indicated good knowledge. For attitudes, a score of 1 was assigned for responses suggesting a positive attitude, and 0 for other responses. Respondents were considered to have a positive overall attitude if they scored ≥ 60%. Practice scores assigned 1 to “always” responses; other responses scored 0. “Not in my scope of practice” responses were excluded. Practice scores ≥ 60% were classified as good.^25^ The internal consistency of the KAP scales was assessed using Cronbach’s Alpha, with values between .7–.95 considered acceptable; however, values below .7 may be secondary to a low number of items or multiple underlying factors.^26^

Bivariate analysis using the Pearson χ^2^ test of Independence (*P* < .05) was conducted to identify associations between knowledge scores and respondent variables. Barriers and facilitators were grouped thematically and presented in frequency tables.

FGD recordings were transcribed verbatim using Microsoft Transcribe. Thematic analysis was conducted following a 6-phase approach, which includes data familiarization, generating initial codes, searching for themes, reviewing themes, defining and naming themes, and producing the report.^27^ Coding was performed using Atlas.ti Version 23.1.1,^28^ and themes were organized in Microsoft Word.

### Ethical considerations

Ethical approval was obtained from NUST and MoHSS. Informed consent was obtained from all participants. The online questionnaire contained an information sheet and a tick-box for digital consent (Supplemental File 1), while FDG participants provided written informed consent (Supplemental File 2). Data was anonymised and stored securely on a password-protected device, with access limited to the researchers.

## Results

### Quantitative phase

#### Respondent characteristics

Of the 102 responses, 62.7% (n = 64) were female, 63.7% (n = 65) were between 30 and 39 years, 62.7% (n = 64) worked in hospitals, and 42.2% (n = 43) had 6–10 years’ work experience (Table 1). A certificate (National Qualifications Framework [NQF] level 5) was the highest qualification for 29 (28.4%) respondents, while 26 (25.5%) had an honors, professional degree, or postgraduate diploma (NQF level 8). Most respondents were nurses (n = 44, 43.1%), with a similar distribution of responses observed per district. Only 32 (31.4%) have been vaccinated against TB, while 12 (11.8%) have been previously infected with TB. Less than half (n = 46, 45.1%) of respondents received TB IPC training in the last 2 years.

**Table 1.** Respondent characteristics

Respondent characteristics	n (%)
**Gender**	
Male	38 (37.3%)
Female	64 (62.7%)
**Age distribution**	
20–29 years	27 (26.5%)
30–39 years	65 (63.7%)
40–49 years	7 (6.9%)
≥ 50 years	3 (2.9%)
**District**	
Keetmanshoop	30 (29.4%)
Karasburg	38 (37.3%)
Lüderitz	34 (33.3%)
**Type of health facility**	
Hospital	64 (62.7%)
Clinic	30 (29.4%)
Health center	4 (3.9%)
Other	4 (3.9%)
**Current role**	
Nurse	44 (43.1%)
Health assistant/ward attendant	24 (23.5%)
Environmental health practitioner/assistant	8 (7.8%)
Lab technologist/technician	4 (3.9%)
Medical doctor (MD)	3 (2.9%)
Radiographer/X-ray technician	2 (2.0%)
Other	17 (16.7%)
**Department**	
Casualty/emergency department	4 (3.9%)
General ward	9 (8.8%)
HIV care department	11 (10.8%)
Laboratory	4 (3.9%)
MDR-TB ward	23 (22.5%)
Outpatient department	13 (12.7%)
Radiology/X-ray	2 (2.0%)
Other	36 (35.3%)
**Highest education**	
Grade 12 (matric)	6 (5.9%)
Certificate (NQF level 5)	29 (28.4%)
Diploma (NQF level 6)	22 (21.6%)
Degree (NQF level 7)	18 (17.6%)
Honors, professional degree, or postgraduate diploma (NQF level 8)	26 (25.5%)
Master’s degree (NQF level 9)	1 (1.0%)
PhD or doctorate (NQF level 10)	0 (.0%)
**Experience in current position**	
6 months–1 year	8 (7.8%)
1–5 years	40 (39.2%)
6–10 years	43 (42.2%)
10–15 years	8 (7.8%)
15–20 years	1 (1.0%)
> 20 years	2 (2.0%)
**Vaccinated against TB**	
Yes	32 (31.4%)
No	70 (68.6%)
**Previously infected with TB**	
Yes	12 (11.8%)
No	90 (88.2%)
**Received TB IPC training in the last 2 years**	
Yes	46 (45.1%)
No	56 (54.9%)
**Total**	**102 (100.0%)**

Note. HIV, human immunodeficiency virus; IPC, infection prevention and control; MDR, multidrug resistance; NQF, National Qualifications Framework; TB, tuberculosis.

#### TB IPC knowledge, attitudes, and practices

The mean knowledge score was 7.4 (SD .77, 95% CI 7.23–7.55) of 10 (74.0%) with 93 (91.2%) respondents obtaining ≥ 60%, suggesting good TB IPC knowledge. The mean score for attitude was 8.3 (SD 1.24, 95%CI 8.07–8.60) of 10 (83.0%), while 87 respondents (85.3%) demonstrated positive attitudes (≥ 60%). For TB IPC practices, the mean score was 14.9 (SD 2.5, 95% CI 14.2–15.5) of 20 (74.5%). Approximately two-thirds of respondents (n = 63, 61.8%) demonstrated good TB IPC practices (≥ 60%) (according to the scope of practice). Supplemental File 3 illustrates responses to the TB IPC KAP statements. Cronbach’s alpha for the knowledge, attitudes, and practice sections was .57, .17, and .87, respectively.

#### Characteristics associated with TB IPC knowledge

The Pearson χ^2^ test for Independence was conducted to identify associations between respondent characteristics and knowledge (good or poor). A significant association was found with district (*P* = .001), education level (*P* = .010), and department (*P* = .036). Respondents from the Karasburg (n = 32, 36.5%) and Lüderitz (n = 30, 35.2%) districts appeared more knowledgeable than those from Keetmanshoop (n = 24, 28.3%), while respondents with NQF 5 and 8 qualifications were likewise more knowledgeable than others. Moreover, respondents from the MDR-TB ward (n = 20, 23.2%) and those in “other” departments (n = 33, 38.1%) were more knowledgeable than those working in the general ward, casualty, HIV care, laboratory, outpatient, and radiology departments.

#### Barriers and facilitators to TB IPC adherence

Respondents reported 18 barriers and 13 facilitators to TB IPC adherence. Staff deficit was the main barrier (n = 47, 29.2%), and training (n = 20, 22.5%) was the main facilitator reported (Table 2).

**Table 2.** Barriers and facilitators to TB IPC adherence

Barriers	n (%)
Staff deficit	47 (29.2%)
Lack of TB IPC knowledge	21 (13.0%)
Stigma	5 (3.1%)
Lack of adherence to wearing PPE	3 (1.9%)
Increased number of patients in waiting areas	3 (1.9%)
Negative attitudes and practices toward TB IPC	14 (8.7%)
Training deficit	10 (6.2%)
Lack of resources	29 (18.0%)
Inadequate infrastructure	10 (6.2%)
Lack of management support	8 (5.0%)
Lack of equipment maintenance	1 (.6%)
Uncomfortable PPE	1 (.6%)
Poor staff morale	3 (1.9%)
Not involving all HCWs in TB programs	1 (.6%)
Lengthy guidelines	1 (.6%)
Lack of TB screening of HCWs	1 (.6%)
No established TB IPC committee	2 (1.2%)
Healthcare workers fatigue	1 (.6%)
**Total**	**161 (100.0%)**
**Facilitators**	***n* (%)**
Health promotion and education	12 (13.5%)
Training	20 (22.5%)
Adequate staff	5 (5.6%)
Adequate screening facilities	2 (2.2%)
Good attitudes toward TB IPC	3 (3.4%)
Medicine optimization	1 (1.1%)
Good IPC practices	13 (14.6%)
TB IPC guidelines	2 (2.2%)
Adequate resources	18 (20.2%)
Staff screening	3 (3.4%)
Teamwork	2 (2.2%)
Prompt communication	6 (6.7%)
Monitoring and evaluation	2 (2.2%)
**Total**	**89 (100.0%)**

Note. HCW, healthcare worker; IPC, infection prevention and control; PPE, personal protective equipment; TB, tuberculosis.

## Qualitative phase

### Participant characteristics

Eighteen participants participated in the 3 FGDs. Most participants were nurses (n = 4, 22.2%) in supervisory positions, environmental health practitioners (n = 5, 27.8%), DTLCs (n = 2, 11.1%), and PHC supervisors (n = 2, 11.1%). Ten (55.6%) participants were female, while 44.4% (n = 8) were between 30 and 39 years, and two-thirds practiced for >5 years (n = 12, 66.7%) (Table 3).

**Table 3.** Characteristics of participants (n = 18)

Participant characteristics	Karasburg, n (%)	Lüderitz, n (%)	Keetmanshoop, n (%)	Total, n (%)
**Sex**				
Male	2 (25.0%)	4 (50.0%)	2 (25.0%)	8 (44.4%)
Female	3 (30.0%)	2 (20.0%)	5 (50.0%)	10 (55.6%)
**Age in years**				
20–29 years	0 (.0%)	3 (60.0%)	2 (40.0%)	5 (27.8%)
30–39 years	3 (37.5%)	2 (25.0%)	3 (37.5%)	8 (44.4%)
40–49 years	0 (.0%)	1 (33.3%)	2 (66.7%)	3 (16.7%)
> 50 years	2 (100.0%)	0 (.0%)	0 (.0%)	2 (11.1%)
**Profession**				
DTLC	0 (.0%)	1 (50.0%)	1 (50.0%)	2 (11.1%)
PHC supervisor	1 (50.0%)	1 (50.0%)	0 (.0%)	2 (11.1%)
Medical doctor	0 (.0%)	1 (100.0%)	0 (.0%)	1 (5.6%)
Nurse	0 (.0%)	1 (25.0%)	3 (75.0%)	4 (22.2%)
EHP	2 (40.0%)	1 (20.0%)	2 (40.0%)	5 (27.8%)
Laboratory scientist	0 (.0%)	1(100.0%)	0 (.0%)	1 (5.6%)
DFS	0 (.0%)	0 (.0%)	1 (100.0%)	1 (5.6%)
DS officer	1 (100.0%)	0 (.0%)	0 (.0%)	1 (5.6%)
TB field supervisor	1 (100.0%)	0 (.0%)	0 (.0%)	1 (5.6%)
**Department**				
TB	1 (33.3%)	1 (33.3%)	1 (33.3%)	3 (16.7 %)
ART clinic	0 (.0%)	0 (.0%)	1 (100.0%)	1 (5.6%)
General ward	0 (.0%)	1 (50.0%)	1 (50.0%)	2 (11.1%)
Other	4 (33.3%)	4 (33.3%)	4 (33.3%)	12 (66.7%)
**Practice period**				
Less than 5 years	2 (33.3%)	2 (33.3%)	2 (33.3%)	6 (33.3%)
5–10 years	1 (14.3%)	3 (42.9%)	3 (42.9%)	7 (38.9 %)
More than 10 years	2 (40.0%)	1 (20.0%)	2 (40.0%)	5 (27.8 %)
**Total**	**5 (27.8%)**	**6 (33.3%)**	**7 (38.9%)**	**18 (100.0%)**

Note. ART, anti-retroviral treatment; DFS, district field supervisor; DS, disease surveillance; DTLC, District TB and Leprosy Coordinator; EHP, environmental health practitioner; PHC, primary health care; TB, tuberculosis.

Four themes emerged from the data:

1. Namibia’s effort to fight TB.
2. Healthcare challenges: Exploring TB IPC implementation and adherence barriers.
3. Catalyst for change: Facilitators in the implementation and adherence to TB IPC measures.
4. Improving TB IPC for a healthier tomorrow.

#### Theme 1: Namibia’s effort to fight TB (Table 4)

Participants described a strong commitment to fighting TB through clear IPC measures involving isolation (Q1), triaging and masking patients (Q2), using administrative strategies like FAST (Finding TB cases Actively, Separating safely, Treating effectively) and contact tracing (Q3), ensuring laboratory safety with biosafety cabinets (Q4), and consistent use of PPE (Q5).

**Table 4.** Quotations supporting Themes 1 and 2

No.	Quotations
Q1	*“All the suspected TB patients are being isolated. They have their own rooms until the results come out” (P2, Lüderitz District).*
Q2	*“We do patient triaging at the clinic… we provide them with surgical masks, especially those with signs and symptoms of TB” (P3, Lüderitz District).*
Q3	*“The environmental health department and the TB field promoters do contact tracing as soon as a patient test positive …” (P4, Lüderitz District).*
Q4	*“All specimens are contained in a biosafety cabinet that ensures that your working environment in terms of circulation is safe” (P4, Lüderitz District).*
Q5	*“We always use PPE such as a lab coat, gloves, and N95 masks when handling such specimens in the laboratory” (P5, Lüderitz District).*
Q6	*“The hospital and clinic setup is not designed in such a way to ensure proper ventilation, especially in the general ward” (P1, Lüderitz District).*
Q7	*“Transport is a big issue, especially if we have to pick up patients from the farm or go house to house for health education …” (P4, Karasburg District).*
Q8	*“TB is still considered a discriminatory and stigmatised condition … we are working to screen all our staff members” (P1, Keetmanshoop District).*
Q9	*“Staff shortage is a real barrier to adherence, especially in the hospital … That shortage is a real problem even at the clinics” (P2, Karasburg District).*
Q10	*“Patients who do not want to adhere to the medication or IPC measures are a challenge” (P5, Lüderitz District).*

#### Theme 2: Healthcare challenges: exploring TB IPC implementation and adherence barriers (Table 4)

Participants highlighted barriers such as poor infrastructure (Q6), lack of transport and resources (Q7), persistent stigma in communities (Q8), staff shortages affecting workload and policy implementation (Q9), and patient non-adherence to treatment as well as non-compliance by some HCWs (Q10).

#### Theme 3: Catalyst for change: facilitators in the implementation and adherence to TB IPC measures (Table 5)

Facilitators included effective communication when TB cases are identified (Q11), community and patient health education (Q12), adherence to good TB IPC practices such as consistent mask use and quarterly screening (Q13), sufficient availability of PPE (Q14), and HCW training and education on TB IPC policies (Q15).

**Table 5.** Quotations supporting Themes 3 and 4

No.	Quotations
Q11	*“We also communicate effectively if there is a TB case or a suspected TB case that contact tracing can be done immediately” (P2, Lüderitz District).*
Q12	*“Our patients are well informed on the precautionary measures to prevent the spread of TB” (P1, Keetmanshoop District).*
Q13	*“Healthcare workers wear masks, especially those in the TB ward …We screen our staff members for TB on a quarterly basis” (P2, Karasburg District).*
Q14	*“We have sufficient PPE, such as masks and gloves, at our facility” (P2, Lüderitz District).*
Q15	*“Healthcare workers are educated on TB IPC policies and given those TB IPC guidelines” (P1, Keetmanshoop District).*
Q16	*“We really need to improve our hospital and clinic setup … adequate ventilation … The TB ward also needs to be expanded” (P5, Lüderitz District).*
Q17	*“All vacant posts should be filled … TB field promoters and health assistants should be made permanent …” (P3, Lüderitz District).*
Q18	*“We need more training on IPC in general … Strengthen the health education to the patients and healthcare workers” (P2, Karasburg District).*
Q19	*“We need to focus on informing people that it does not necessarily mean that if somebody has TB then they also have HIV or vice versa” (P6, Karasburg District).*
Q20	*“I think we can strictly monitor the use of PPE when in the TB ward or when working with TB patients …” (P1, Keetmanshoop District).*

#### Theme 4: Improving TB IPC for a healthier tomorrow (Table 5)

Participants suggested strategies for improvement, including upgrading infrastructure for improved ventilation and space (Q16), hiring and retaining sufficient staff (Q17), strengthening training and health education (Q18), addressing stigma and misinformation (Q19), and monitoring PPE usage to ensure compliance (Q20).

## Discussion

The study findings revealed high levels of TB IPC knowledge (91.2%) and attitudes (85.3%), but less optimal practices (61.8%), suggesting a disconnect between TB IPC knowledge and practices of HCWs in the region. Staff deficit and training were the most reported barriers and facilitators, respectively. Conversely, the FGDs revealed that while TB IPC policies existed, their implementation was inconsistent, due to contextual barriers including limited infrastructure, staff shortages, stigma, and inconsistent training. Facilitators included effective communication, health education, and PPE availability.

The TB IPC knowledge observed in this study aligns with findings from Nigeria^25^ but contrasts with studies from Uganda and Namibia reporting poorer knowledge levels. ^8,9^ Disparities may be attributed to differences in knowledge scoring cut-offs and respondent profiles across studies. Consistent with another study,^9^ respondents with certificate-level (NQF 5) and honors/professional degree/postgraduate diploma (NQF 8) qualifications demonstrated higher knowledge scores. This may have been attributed to specifically tailored training in TB IPC and hands-on experience through training or work experiences, reinforcing their knowledge. District-level variances also emerged, with Karasburg respondents showing higher knowledge levels, which again might be linked to level of education.

The study revealed predominantly positive attitudes toward TB IPC, echoing other studies.^8,29^ In contrast, negative attitudes have been shown not to foster TB IPC behavior, hindering administrative and environmental TB IPC compliance.^30^

The implementation of TB IPC measures in public health facilities in //Karas region showed variation. Compared to environmental and PPE control measures, most administrative control measures were poorly implemented. Nevertheless, the current study revealed good overall TB IPC practices. Moreover, the gap between the KAP may be attributable to structural barriers faced in public health settings, such as high workloads, possibly leading to the deprioritization of key administrative controls such as patient education, screening, and triaging. Similarly, poor TB IPC implementation has been documented in Mozambique and South Africa.^31,32^

Despite the existence of TB IPC policies, some HCWs were uninformed about or lacked access to the guidelines. These findings align with previous research indicating poorly disseminated TB IPC guidelines in health facilities.^33^ Moreover, the fragmentation of TB IPC content limited to specific programs hampers comprehensive adherence.^34^ This highlights the importance of having consistent, clearly structured, and widely disseminated guidelines that all HCWs can access and apply.

The qualitative findings also support the global TB IPC framework, which emphasizes the hierarchy of administrative, environmental, and personal protective measures.^5^ However, administrative measures remained the weakest identified component in this study.

Several barriers impeded the effective implementation of TB IPC measures, including critical human resource shortages, high patient workload, and inadequate infrastructure, factors that have similarly been reported in other high TB-burden settings.^35^ Poor coordination between stakeholders, especially at district levels, and inconsistent availability of TB IPC guidelines further undermine standardized implementation.^12^

Despite these challenges, key facilitators supported TB IPC efforts. These included routine training, the appointment of dedicated focal persons, and the existence of national TB IPC policies, which were widely implemented across facilities. The presence of multidisciplinary TB IPC committees and supportive supervision further contributed to strengthening facility-level implementation, even in resource-constrained environments.^36^

The study was limited to the //Karas region, restricting generalizability. Adding additional regions in future research would add further insight. Participant reluctance may have also influenced responses despite being assured of anonymity and confidentiality. Moreover, the questionnaire received a low response rate, likely due to staff shortages, poor internet connectivity in remote areas, and possible survey fatigue, which may introduce non-response bias and limit representativeness. Consequently, the findings may not fully represent all HCWs in the region, and key measures such as TB IPC knowledge or adherence may be over- or underestimated. To improve participation, the questionnaire link was shared via social media along with periodic reminders. Furthermore, the absence of established IPC committees in most districts may have limited the depth and accuracy of FGD responses. Additionally, the internal consistency of the knowledge and attitude scales was low, suggesting caution when interpreting these results. Despite these limitations, the findings remain applicable to TB focal persons working in public health facilities within the region.

In conclusion, this study provides valuable insight into the KAP of HCWs regarding TB IPC in the //Karas region of Namibia, revealing important gaps in implementation despite widespread policy uptake. Our findings underscore the significance of strengthening HCW capacity through ongoing training, ensuring accessibility of TB IPC guidelines, and fostering interdisciplinary collaboration for effective policy implementation. Priority improvements should focus on improving ventilation and increasing isolation room capacity, as poor infrastructure was a major barrier to effective TB IPC. Other critical barriers to address include staff shortages, inconsistent TB IPC training, limited PPE, and persistent stigma, all of which impede TB IPC adherence. By highlighting these factors, the study contributes to the development of evidence-based TB IPC strategies that can improve IPC in health settings.

Future research should focus on conducting longitudinal studies to track the implementation of TB IPC measures and adherence among HCWs. This could assist in identifying trends, challenges, and variations in behavior over time, allowing for the evaluation of the sustainability of TB IPC practices and the effectiveness of ongoing interventions.

## Supporting information

10.1017/ash.2025.10248.sm001 Nyambe et al. supplementary material 1 Nyambe et al. supplementary material

10.1017/ash.2025.10248.sm002 Nyambe et al. supplementary material 2 Nyambe et al. supplementary material

10.1017/ash.2025.10248.sm003 Nyambe et al. supplementary material 3 Nyambe et al. supplementary material

## Supplementary material

The supplementary material for this article can be found at https://doi.org/10.1017/ash.2025.10248
