# Supplementary material for: TB infection prevention and control at public health facilities in //Karas region, Namibia
Source: Antimicrob Steward Healthc Epidemiol. 2025 Dec 16;5(1):e341. doi: 10.1017/ash.2025.10248 (PMC12722535; doi:10.1017/ash.2025.10248)
Supplement: Nyambe et al. supplementary material 1 — Nyambe et al. supplementary material [file S2732494X25102489sup001.docx]

**Supplemental File 1:** Questionnaire

**TITLE OF RESEARCH PROJECT:** TB Infection Prevention and Control at public health facilities in //Karas region, Namibia.

**SECTION A: INFORMED CONSENT**

**DETAILS OF THE RESEARCHER:**

**Name:** Nicolett Nyambe

**Occupation:** Environmental Health Practitioner

**Qualification:** B.Sc Environmental Health Sciences

Contact details: cell: +264816447271, email: [njnyambe@gmail.com](mailto:njnyambe@gmail.com)

**The research objectives:**

1. To evaluate the TB IPC knowledge, attitudes, and practices (KAP) of HCWs providing TB services at public health facilities in //Karas region.
2. To assess the barriers and facilitators to the implementation and adherence to TB IPC procedures at public health facilities in //Karas region.
3. To evaluate policies aimed at TB IPC at public health facilities in //Karas region.

You are invited to take part in a research project which involves the completion of an online questionnaire. Your participation is entirely voluntary and will be highly appreciated but feel free to decline. Refusal to parttake in the survey will not affect you professionally in any way.  Please note that once you submit your completed online questionnaire, it will be anonymised and you will no longer be able to withdraw your responses. The research is conducted by Ms. Nicolett Nyambe who is a Master of Health Sciences student at the Namibia University of Science and Technology (NUST). This online questionnaire is the first phase (quantitative) of a mixed-method study. You will not be required to participate in the next phase of the study.

The study invites different cadres of healthcare workers such as medical doctors, nurses, health assistants/ward attendants, radiographers/x-ray technicians, environmental health practitioners/assistants, Laboratory technologists/technicians and other healthcare workers providing TB services at public health facilities in //Karas region working at their current healthcare facility for more than 6 months. The study will exclude TB healthcare workers with less than 6 months experience at their current healthcare facility, those on leave during the data collection period and hospital staff involved in administration and management.

There are no potential benefits or risks from taking part in this research. This research is strictly for academic purposes.

**Anonymity****:** Your identity will not be used in this study. Therefore, please do not record names in the questionnaire.

**Confidentiality**: Data will be saved and encrypted in the researcher’s computer file (password protected) which will only be accessible by the researcher and the supervisors.

**Ethical approval:** This study received ethical clearance and permission to conduct research from the NUST Ethics Committee and the Ministry of Health and Social Services.

For more information on the research please feel free to contact the researcher of this study Ms. Nicolett Nyambe on the contact details given above.

By clicking **YES**, you are confirming that you have:

- **Read and understood the information above.**
- **You agree to participate in the study (strictly voluntary).**

By clicking **NO**, you are confirming that you are not taking part in the study.

**Duration: approximately 20 minutes**

**Do you wish to take part in this study?**

- Yes
- No

**SECTION B: Socio-demographic Characteristics of Healthcare Workers**

This section contains 10 questions on the socio-demographic characteristics of healthcare workers.

1. **What is your current role?**

- Medical Doctor (MD)
- Nurse
- Health assistant/ward attendant
- Radiographer/X-ray technician
- Environmental health practitioner/assistant
- Laboratory technologist/technician
- Other (Please specify below)

**If ‘other’ selected above (in question 1), please specify below.**

………………………………………………………………………………………………..

1. **What is your gender?**

- Male
- Female

1. **What is your age (in years)?**

- 20-29 years
- 30-39 years
- 40-49 years
- >50 years

1. **What district are you currently working in?**

- Keetmanshoop district
- Luderitz district
- Karasburg district

1. **What type of healthcare facility are you based at?**

- Hospital
- Clinic
- Health centre
- Other (Please indicate below)

**If ‘other’ selected above (in question 5), please specify below.**

………………………………………………………………………………………………..

1. **What department are you working in?**

- Outpatient department
- Casualty/Emergency department
- Radiology/ X-ray department
- MDR-TB ward
- HIV care department
- General ward
- Laboratory
- Other (Please indicate below)

**If ‘other’ selected above (in question 6), please specify below.**

………………………………………………………………………………………………..

1. **Indicate your highest level of education.**

- Grade 12 (Matric)
- Certificate (NQF level 5)
- Diploma (NQF level 6)
- Degree (NQF level 7)
- Honours, Professional Degree or Postgraduate Diploma (NQF level 8)
- Master’s degree (NQF level 9)
- PhD or Doctorate (NQF level 10)

1. **Indicate the years of experience in your current profession.**

- 6 months-1 year
- 1-5 years
- 6-10 years
- 10-15 years
- 15-20 years
- >20 years

1. **Have you ever been infected with TB?**

- Yes
- No

1. **Are you vaccinated against TB (BSG vaccine??**

- Yes
- No

**SECTION C: TB IPC Training**

1. **Have you received training on TB Infection Prevention and Control (IPC) in the last 2 years.**

- Yes
- No

**SECTION D: TB IPC Knowledge of HCWs**

This section contains 10 questions on TB Infection Prevention and Control knowledge. For each of the following questions, choose the one (True/Don’t know/False) that you understand to be appropriate.

1. **TB is an airborne bacterial infection caused by *mycobacterium tuberculosis* (M. tuberculosis).**

- True
- Don’t know
- False

1. **TB is spread by airborne transmission when the TB bacteria is expelled into the air in tiny droplets by a person with active TB disease.**

- True
- Don’t know
- False

1. **A person with active TB disease usually has a persistent cough that lasts more than 3 weeks.**

- True
- Don’t know
- False

1. **A person with Latent TB infection does not show any signs or symptoms of TB.**

- True
- Don’t know
- False

1. **The risk of transmission increases with close and prolonged contact with an infectious TB patient.**

- True
- Don’t know
- False

1. **The standard treatment for drug-sensitive pulmonary TB is 3 months.**

- True
- Don’t know
- False

1. **The main diagnostic tool for pulmonary TB in Namibia is the sputum smear microscopy.**

- True
- Don’t know
- False

1. **Triage of people with TB signs and symptoms does not reduce TB transmission in a healthcare setting.**

- True
- Don’t know
- False

1. **Sufficient natural and artificial ventilation systems reduce the concentration of infectious airborne pathogens.**

- True
- Don’t know
- False

1. **N95 masks can protect healthcare workers from inhaling infectious aerosols.**

- True
- Don’t know
- False

**SECTION E: Attitude of HCWs towards TB IPC**

This section contains 10 questions on TB infection Prevention and Control attitude. For each of the following questions, choose the one (1= Agree, 2=Neutral, 3= Disagree) that you understand to be appropriate.

1. **Healthcare facilities should have TB infection prevention and control policies in place.**

| 1 – Agree | 2 - Neutral | 3 – Disagree |
| --- | --- | --- |

1. **Healthcare workers should be trained in TB infection prevention and control policies.**

| 1 – Agree | 2 - Neutral | 3 – Disagree |
| --- | --- | --- |

1. **Healthcare workers who show symptoms suggestive of TB should not be screened.**

| 1 – Agree | 2 - Neutral | 3 – Disagree |
| --- | --- | --- |

1. **Changing surgical gloves is not necessary during procedures even if heavily contaminated.**

| 1 – Agree | 2 - Neutral | 3 – Disagree |
| --- | --- | --- |

1. **TB can be treated hence personal protective equipment are not required.**

| 1 – Agree | 2 - Neutral | 3 – Disagree |
| --- | --- | --- |

1. **Healthcare workers must ensure that surfaces and medical equipment are adequately disinfected.**

| 1 – Agree | 2 - Neutral | 3 – Disagree |
| --- | --- | --- |

1. **Healthcare workers may turn off fans or close windows in the TB ward if it becomes too cold.**

| 1 – Agree | 2 - Neutral | 3 – Disagree |
| --- | --- | --- |

1. **It is uncomfortable to work with personal protective equipment in the healthcare facility.**

| 1 – Agree | 2 - Neutral | 3 – Disagree |
| --- | --- | --- |

1. **Both washing and disinfecting hands after handling TB patients are not needed.**

| 1 – Agree | 2 - Neutral | 3 – Disagree |
| --- | --- | --- |

1. **Sputum induction procedures should only be performed by trained healthcare workers.**

| 1 – Agree | 2 - Neutral | 3 – Disagree |
| --- | --- | --- |

**SECTION F: Practices of HCWs towards TB IPC**

This section contains 11 questions on TB Infection Prevention and Control practices. For each of the following questions, choose the one that you understand to be appropriate.

**Administrative control measures**

1. **How often do you educate patients and co-workers about TB?**

- Always
- Sometimes
- Never
- Not in my scope of practice

1. **How often do you give respiratory hygiene education/cough etiquette to TB patients?**

- Always
- Sometimes
- Never
- Not in my scope of practice

1. **How often do you screen suspicious TB patients in waiting areas?**

- Always
- Sometimes
- Never
- Not in my scope of practice

1. **How often do you triage patients with TB signs and symptoms?**

- Always
- Sometimes
- Never
- Not in my scope of practice

1. **How often do you wash your hands? (choose the options applicable to your current role).**

|  | Always | Sometimes | Never | Not scope of practice |
| --- | --- | --- | --- | --- |
| After coming in contact with TB patients. |  |  |  |  |
| After handling sputum samples. |  |  |  |  |
| After visiting TB ward. |  |  |  |  |
| After performing a chest radiography. |  |  |  |  |

1. **How often do you conduct TB risk assessment at your healthcare facility?**

- Always
- Sometimes
- Never
- Not in my scope of practice

**Environmental Control Measures**

1. **How often do you open windows for ventilation in your specific department?**

- Always
- Sometimes
- Never
- Not in my scope of practice

1. **How often do you ensure that sputum collection from a patient is in a separate, well-ventilated area or outdoor?**

- Always
- Sometimes
- Never
- Not in my scope of practice

**Personal Protection Measures**

1. **How often do you provide a surgical mask to a suspected TB patient?**

- Always
- Sometimes
- Never
- Not in my scope of practice

1. **How often do you wear an N95 mask (choose the options applicable to your current role)?**

|  | Always | Sometimes | Never | Not scope of practice |
| --- | --- | --- | --- | --- |
| After coming in contact with TB patients. |  |  |  |  |
| After handling sputum samples. |  |  |  |  |
| After visiting TB ward. |  |  |  |  |
| After performing a chest radiography. |  |  |  |  |

1. **How often do you use surgical gloves (choose the options applicable to your current role)?**

|  | Always | Sometimes | Never | Not scope of practice |
| --- | --- | --- | --- | --- |
| After coming in contact with TB patients. |  |  |  |  |
| After handling sputum samples. |  |  |  |  |
| After visiting TB ward. |  |  |  |  |
| After performing a chest radiography. |  |  |  |  |

**SECTION G: Barriers and facilitators to adherence to TB IPC procedures.**

In the spaces provided, please indicate any barriers and facilitators to the adherence to TB IPC procedures you have experienced at your healthcare facility (please add all barriers and facilitators you have experienced). Barriers refer to obstacles/factors that prevent you from achieving something while facilitators are factors that enable you to achieve something.

1. **What in your opinion are the barriers to the adherence to TB infection and control at your healthcare facility?**

…………………………………………………………………………………………………………

1. **What in your opinion are the facilitators to the adherence to TB infection and control at your healthcare facility?**

………………………………………………………………………………………………………

**Thank you for participating in this study**
