# Supplementary material for: TB infection prevention and control at public health facilities in //Karas region, Namibia
Source: Antimicrob Steward Healthc Epidemiol. 2025 Dec 16;5(1):e341. doi: 10.1017/ash.2025.10248 (PMC12722535; doi:10.1017/ash.2025.10248)
Supplement: Nyambe et al. supplementary material 2 — Nyambe et al. supplementary material [file S2732494X25102489sup002.docx]

**Supplementary** **File 2:** Focus Group Discussion Guide

**WELCOMING REMARKS**

I would like to welcome you to the focus group discussion. Thank you for taking the time to take part in the discussion about TB Infection Prevention & Control (IPC) policies in //Karas region. My name is Nicolett Nyambe, I am a Master of Health Science Student at the Namibia University of Science and Technology (NUST). Currently, I am doing a study on TB Infection Prevention and Control at public health facilities in //Karas region, Namibia.

You have been chosen to participate because you are key persons to discuss information on the Infection Prevention and Control (IPC) policies aimed at TB IPC in the //Karas region. Your input on the topic will be highly appreciated. Therefore, please feel free to engage in the discussion honestly. I will moderate the discussion myself and the focus group will be audio-recorded, and side notes will be taken.

Name of Healthcare facility/district: ______________________________

Name of Researcher: ____________________________________

Profession of healthcare worker: ___________________________

**Opening Question**

1. Can you briefly provide an outline of the TB IPC policies and guidelines implemented in your healthcare facility?

**Key Questions**

1. How are these TB IPC policies made available to every TB healthcare worker in the healthcare facility?
2. How does your healthcare facility collaborate with national TB programmes and other stakeholders to ensure a coordinated approach to TB IPC?
3. Can you provide insights into the monitoring and evaluation mechanisms in place to assess the implementation and effectiveness of TB IPC policies?
4. What do you perceive to be the barriers to the implementation and adherence to TB infection prevention and control at your healthcare facility?
5. Which infection control practices do you find the most challenging to implement in your healthcare setting and why?
6. Are there any other specific challenges that you would like to highlight in relation to TB IPC implementation?
7. What do you think are the facilitators in the implementation and adherence to TB infection prevention and control at your healthcare facility?
8. What are your preferred approaches or strategies to improve TB IPC practices in your healthcare setting?
9. How do you manage healthcare-acquired infections in the healthcare setting?
10. Are there any final opinions or experiences you wish to share?

**Thank you for your time**
