# Supplementary material for: TB infection prevention and control at public health facilities in //Karas region, Namibia
Source: Antimicrob Steward Healthc Epidemiol. 2025 Dec 16;5(1):e341. doi: 10.1017/ash.2025.10248 (PMC12722535; doi:10.1017/ash.2025.10248)
Supplement: Nyambe et al. supplementary material 3 — Nyambe et al. supplementary material [file S2732494X25102489sup003.docx]

Supplemental File 3: TB IPC Knowledge, Attitudes and Practices of HCWs (n=102)

| **TB IPC Knowledge Variables (n=102)** | **True, n (%)** | **False, n (%)** | **Don’t know, n (%)** |
| --- | --- | --- | --- |
| Q1: TB is an airborne bacterial infection caused by Mycobacterium tuberculosis. | 102 (100.0%) * | 0 (0.0%) | 0 (0.0%) |
| Q2: TB is spread by airborne transmission when the TB bacteria is expelled into the air in tiny droplets by a person with active TB disease. | 101 (99.0%) * | 0 (0.0%) | 1 (1.0%) |
| Q3: A person with active TB disease usually has a persistent cough that lasts more than 3 weeks. | 93 (91.2%) * | 4 (3.9%) | 5 (4.9%) |
| Q4: A person with Latent TB infection does not show any signs or symptoms of TB. | 78 (76.5%) * | 9 (8.8%) | 15 (14.7%) |
| Q5: The risk of transmission increases with close and prolonged contact with an infectious TB patient. | 93 (91.2%) * | 2 (2.0%) | 7 (6.9%) |
| Q6: The standard treatment for drug-sensitive pulmonary TB is 3 months. | 22 (21.6%) | 60 (58.8%) * | 20 (19.6%) |
| Q7: The main diagnostic tool for pulmonary TB in Namibia is the sputum smear microscopy. | 73 (71.6%) * | 8 (7.8%) | 21 (20.6%) |
| Q8: Triage of people with TB signs and symptoms does not reduce TB transmission in a healthcare setting. | 11 (10.8%) | 76 (74.5%) * | 15 (14.7%) |
| Q9: Sufficient natural and artificial ventilation systems reduce the concentration of infectious airborne pathogens. | 89 (87.3%) * | 5 (4.9%) | 8 (7.8%) |
| Q10: N95 masks can protect healthcare workers from inhaling infectious aerosols. | 98 (96.1%) * | 1 (1.0%) | 3 (2.9%) |
| **IPC Attitude Variables (n=102)** | **Agree, n (%)** | **Neutral, n (%)** | **Disagree, n (%)** |
| Q11: Healthcare facilities should have TB infection prevention and control policies in place. | 96 (94.1%) ^†^ | 6 (5.9%) | 0 (0.0%) |
| Q12: Healthcare workers should be trained on TB infection prevention and control policies. | 96 (94.1%) ^†^ | 6 (5.9%) | 0 (0.0%) |
| Q13: Healthcare workers who show symptoms suggestive of TB should not be screened. | 13 (12.7%) | 7 (6.9%) | 82 (80.4%) ^†^ |
| Q14: Changing surgical gloves is not necessary during procedures, even if heavily contaminated. | 4 (3.9%) | 19 (18.6%) | 79 (77.5%) ^†^ |
| Q15: TB can be treated hence personal protective equipment is not required. | 1 (1.0%) | 16 (15.7%) | 85 (83.3%) ^†^ |
| Q16: Healthcare workers must ensure that surfaces and medical equipment are adequately disinfected. | 74 (72.5%) ^†^ | 26 (25.5%) | 2 (2.0%) |
| Q17: Healthcare workers may turn off fans or close windows in the TB ward if it becomes too cold. | 6 (5.9%) | 39 (38.2%) | 57 (55.9%) ^†^ |
| Q18: It is uncomfortable to work with personal protective equipment in the healthcare facility. | 6 (5.9%) | 44 (43.1%) | 52 (51.0%) ^†^ |
| Q19: Both washing and disinfecting hands after handling TB patients are not needed. | 0 (0.0%) | 9 (8.8%) | 93 (91.2%) ^†^ |
| Q20: Sputum induction procedures should only be performed by trained healthcare workers. | 86 (84.3%) ^†^ | 11 (10.8%) | 5 (4.9%) |
| **IPC Practice Variables (Report per practitioner scope of practice)** | **Always, n (%)** | **Sometimes, n (%)** | **Never, n (%)** |
| **Administrative Control Measures** | | | |
| Q21 How often do you educate patients and co-workers about TB (n=99)? | 53 (53.5%)  ^‡^ | 41 (41.4%) | 5 (5.1%) |
| Q22 How often do you give respiratory hygiene education/cough etiquette to TB patients (n=96)? | 50 (52.1%)  ^‡^ | 43 (44.8%) | 3 (3.1%) |
| Q23 How often do you screen suspicious TB patients in waiting areas (n=86)? | 38 (44.2%)  ^‡^ | 43 (50.0%) | 5 (5.8%) |
| Q24 How often do you triage patients with TB signs and symptoms (n=86)? | 45 (52.3%)  ^‡^ | 36 (41.9%) | 5 (5.8%) |
| Q25 How often do you wash hands after coming in contact with TB patients (n=99)? | 81 (81.8%)  ^‡^ | 18 (18.2%) | 0 (0.0%) |
| Q26 How often do you wash hands after handling sputum samples (n=91)? | 77 (84.6%)  ^‡^ | 13 (14.3%) | 1 (1.1%) |
| Q27 How often do you wash hands after visiting TB ward (n=99)? | 73 (73.7%)  ^‡^ | 25 (25.3%) | 1 (1.0%) |
| Q28 How often do you wash hands after performing a chest radiography (n=39)? | 30 (76.9%)  ^‡^ | 4 (10.3%) | 5 (12.8%) |
| Q29 How often do you conduct TB risk assessments at your healthcare facility (n=90)? | 21 (23.3%)  ^‡^ | 47 (52.2%) | 22 (24.4%) |
| **Environmental Control Measures** | | | |
| Q30 How often do you open windows for ventilation in your specific department (n=102)? | 74 (72.5%)  ^‡^ | 27 (26.5%) | 1 (1.0%) |
| Q31 How often do you ensure sputum collection from a patient is in a separate, well-ventilated area or outdoor (n=82)? | 61 (74.4%)  ^‡^ | 18 (22.0%) | 3 (3.7%) |
| **Personal Protective Control Measures** | | | |
| Q32 How often do you provide a surgical mask to a suspected TB patient (n=102)? | 72 (70.6%)  ^‡^ | 28 (27.5%) | 2 (2.0%) |
| Q33 How often do you wear an N95 mask when coming in contact with TB patients (n=100)? | 90 (90.0%)  ^‡^ | 8 (8.0%) | 2 (2.0%) |
| Q34 How often do you wear an N95 mask when handling sputum samples (n=92)? | 77 (83.7%)  ^‡^ | 10 (10.9%) | 5 (5.4%) |
| Q35 How often do you wear an N95 mask when visiting the TB ward (n=99)? | 90 (90.9%)  ^‡^ | 8 (8.1%) | 1 (1.0%) |
| Q36 How often do you wear an N95 mask when performing a chest radiography (n=39)? | 23 (59.0%) ^‡^ | 13 (33.3%) | 3 (7.7%) |
| Q37 How often do you use surgical gloves when coming in contact with TB patients (n=98)? | 77 (78.6 %) ^‡^ | 18 (18.4%) | 3 (3.1%) |
| Q38 How often do you use surgical gloves when handling sputum samples (n=91)? | 87 (96.0%) ^‡^ | 3 (3.3%) | 1 (1.1%) |
| Q39 How often do you use surgical gloves when visiting the TB ward (n=99)? | 76 (76.8%) ^‡^ | 18 (18.2%) | 5 (5.1%) |
| Q40 How often do you use surgical gloves when performing a chest radiography (n=38)? | 22 (57.9%) ^‡^ | 12 (31.6%) | 4 (10.5%) |
| Footnote^: *^ Indicates correct responses for TB IPC knowledge, ^†^ Indicates positive attitude toward TB IPC, ^‡^ Indicates good TB IPC practices. | | | |
